# A Computational Model for Predicting Nanoparticle Accumulation in Tumor Vasculature

**DOI:** 10.1371/journal.pone.0056876

**Published:** 2013-02-28

**Authors:** Hermann B. Frieboes, Min Wu, John Lowengrub, Paolo Decuzzi, Vittorio Cristini

**Affiliations:** 1 Department of Bioengineering, University of Louisville, Louisville, Kentucky, United States of America; 2 James Graham Brown Cancer Center, University of Louisville, Louisville, Kentucky, United States of America; 3 Department of Pathology, University of New Mexico, Albuquerque, New Mexico, United States of America; 4 Department of Mathematics, University of California Irvine, Irvine, California, United States of America; 5 Department of Translational Imaging and Department of Nanomedicine, The Methodist Hospital Research Institute, Houston, Texas, United States of America; 6 Department of Chemical Engineering, University of New Mexico, Albuquerque, New Mexico, United States of America; Case Western Reserve University, United States of America

## Abstract

Vascular targeting of malignant tissues with systemically injected nanoparticles (NPs) holds promise in molecular imaging and anti-angiogenic therapies. Here, a computational model is presented to predict the development of tumor neovasculature over time and the specific, vascular accumulation of blood-borne NPs. A multidimensional tumor-growth model is integrated with a mesoscale formulation for the NP adhesion to blood vessel walls. The fraction of injected NPs depositing within the diseased vasculature and their spatial distribution is computed as a function of tumor stage, from 0 to day 24 post-tumor inception. As the malignant mass grows in size, average blood flow and shear rates increase within the tumor neovasculature, reaching values comparable with those measured in healthy, pre-existing vessels already at 10 days. The NP vascular affinity, interpreted as the likelihood for a blood-borne NP to firmly adhere to the vessel walls, is a fundamental parameter in this analysis and depends on NP size and ligand density, and vascular receptor expression. For high vascular affinities, NPs tend to accumulate mostly at the inlet tumor vessels leaving the inner and outer vasculature depleted of NPs. For low vascular affinities, NPs distribute quite uniformly intra-tumorally but exhibit low accumulation doses. It is shown that an optimal vascular affinity can be identified providing the proper balance between accumulation dose and uniform spatial distribution of the NPs. This balance depends on the stage of tumor development (vascularity and endothelial receptor expression) and the NP properties (size, ligand density and ligand-receptor molecular affinity). Also, it is demonstrated that for insufficiently developed vascular networks, NPs are transported preferentially through the healthy, pre-existing vessels, thus bypassing the tumor mass. The computational tool described here can effectively select an optimal NP formulation presenting high accumulation doses and uniform spatial intra-tumor distributions as a function of the development stage of the malignancy.

## Introduction

The efficacy of conventional chemotherapeutic and imaging agents is impaired mostly by their suboptimal accumulation at the target site, the tumor tissue [1]–[3]. Systemically injected small molecules tend to deposit non-specifically in almost any region perfused by blood, thus reaching the malignant mass at doses generally insufficient to eradicate the disease or enhance the imaging contrast. The application of nanotechnology to biomedical sciences has paved the way for the development of novel strategies applied to the early detection and more efficient treatment of diseases [4]–[6]. In oncology, potent chemotherapeutic molecules have been reformulated into liposomes and nanoparticles (NPs), demonstrating improved pharmacokinetics and pharmacodynamics, and reduced off-target toxicity [7]–[8]. Nonetheless, the dose of active molecules deposited at target sites is still largely suboptimal and in need of improvement.

NPs are man-made objects sufficiently small to circulate safely within the vascular systems while transported by the blood flow [4]–[6]. Their size, shape and surface properties can be tailored during the synthesis process to recognize specifically either a tumor cell within the malignant mass (tumor targeting), or an endothelial cell lining the diseased microvasculature (vascular targeting). In the first case, NPs need to be sufficiently small to passively cross the fenestrations occurring in the discontinuous tumor endothelium and thus take advantage of the well-known Enhanced Permeation and Retention (EPR) effect [9]–[11]. Typically, tumor-targeted particles are spherical with a diameter ranging between ∼50 and 300 nm. However, the size of the tumor fenestrations varies with the type, stage and location of the disease, ranging broadly from 20 nm for brain tumors to a few micrometers in the case of subcutaneous malignancies [1], [11].

In vascular targeting, the surface of the NPs is decorated with molecules (ligands) that can recognize counter-molecules (receptors) specifically expressed or over-expressed on the membrane of the tumor endothelial cells. Over the last decade, potent technologies have been developed to identify and quantify, even in humans, endothelial receptor molecules expressed in several vascular regions [12]–[13]. The size of the vascularly-targeted NPs is not limited by the endothelial fenestrations and can be as large as a few microns. This strategy, therefore, allows for a larger amount of drug molecules and contrast agents to be delivered to the diseased vessels in the pursuit of anti-angiogenic therapies and early disease detection. In previous work we have shown that size, shape and surface properties of NPs can be optimized to enhance the recognition of specific vascular targets and favor stable, firm adhesion under flow [14]–[16].

Since the transport of any systemically injected agent to the malignant tissue is dictated by the tumor vasculature [17]–[18], it is reasonable to speculate that the tumoritropic accumulation of NPs could be affected by the stage of development of the vessel network and concomittant expression of vascular receptor molecules. To verify this hypothesis and optimize the tumoritropic accumulation of NPs, here we develop a multidimensional computational model to predict the accumulation of systemically injected NPs in tumors. This is obtained by implementing a mesoscale model for the vascular adhesion of NPs [15] with a multi-dimensional tumor growth model that links cellular-level events to the tumor tissue scale while accounting for the time-dependent development of the tumor-induced vasculature [19]–[23]. The spatial distribution and fraction of NPs depositing within the diseased vasculature is computed as a function of the tumor size, biophysical properties of the tumor vessel walls, and NP features. During the evolution of the malignant mass from the time of inception to day 24, the tumor induced vascular network is characterized in terms of vessel density, perfusion and shear rates. Spherical NPs are considered with a diameter ranging from 100 to 1,000 nm. This computational model system enables to predict the tumoritropic accumulation of NPs depending on the tumor stage.

## Results

### Simulation of Particle Transport

**Figure 1:**
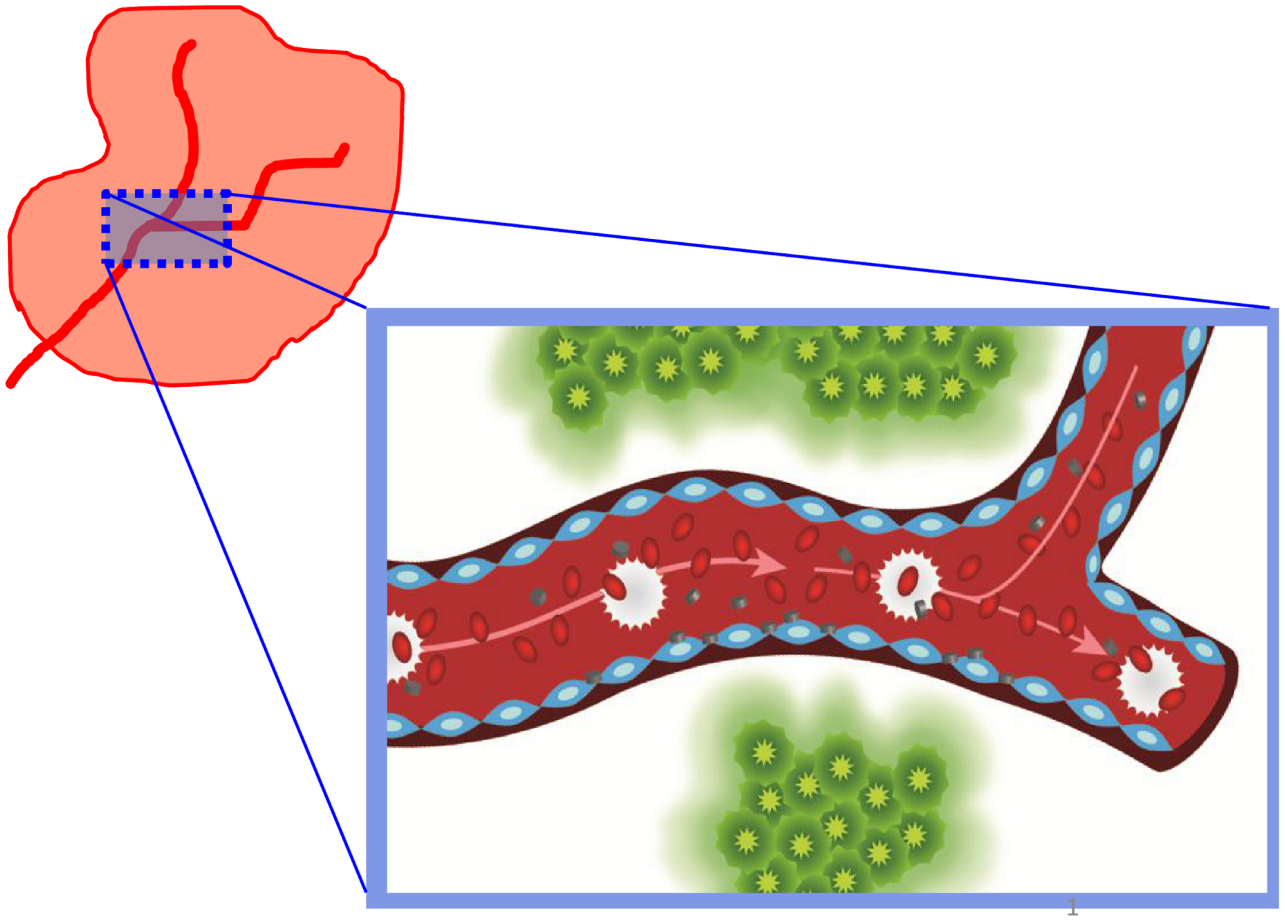
Highlight of a blood vessel network lined by endothelial cells (blue) transporting red blood cells (red), leukocytes (white) and nanoparticles (grey) within a tumor. Some of the nanoparticles are shown firmly adhering to the endothelial cells while experiencing intra-vascular flow.

We simulate the transport and progressive accumulation of NPs within the tumor microvasculature, as schematically shown in **Figure 1**. The systemically injected NPs reach the malignant mass through the pre-existing vascular network and the more chaotic neovasculature originating over time within the tumor. Spherical NPs with three different sizes, namely 100, 600 and 1,000 nm, are considered. The surface density and molecular affinity of the ligand molecules decorating the NPs, as well as the receptor molecules expressed on the tumor endothelium, are systematically varied through the parameters α and β, as described in the **Methods**. The objective of this analysis is to characterize variations in the vascular accumulation of NPs depending on the tumor development stage and the expression of vascular endothelial receptors.

### Tumor Development

**Figure 2:**
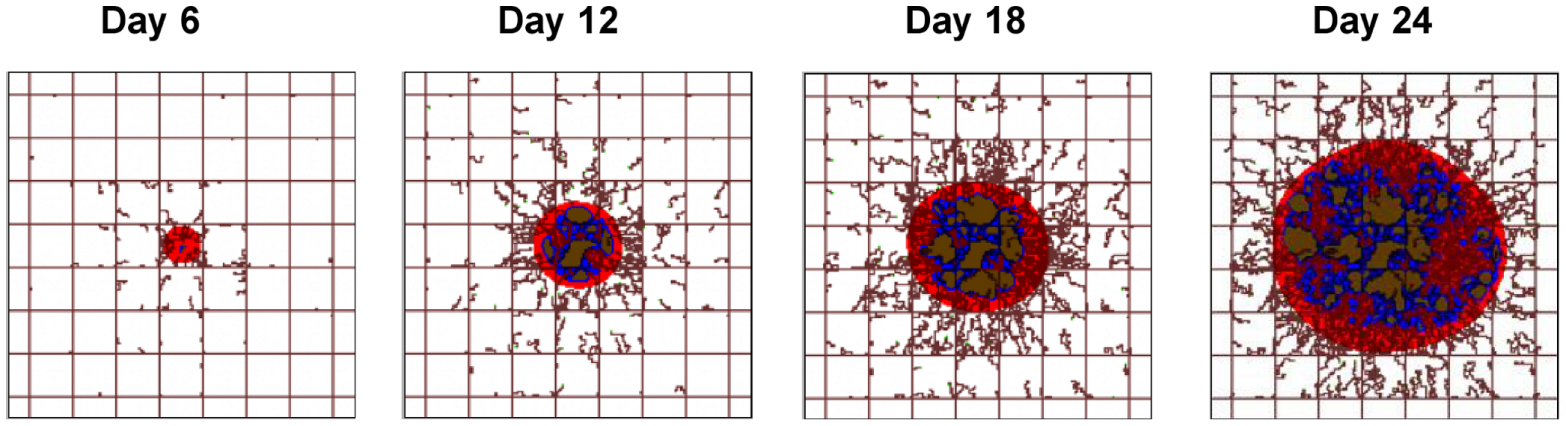
The progressive development of the malignant mass is depicted at four different time points, namely 6, 12, 18 and 24 days post tumor inception. Three characteristic tumor regions can be identified as the viable (red), hypoxic (blue), and necrotic (brown) tissue. Pre-existing vessels (straight brown lines) are laid out in a regular grid, maintaining normoxic conditions in the surrounding tissue. New vessels (irregular brown lines) are sprouting from the pre-existing vasculature in response to a net balance of pro-angiogenic factors released by hypoxic cells in the interior of the tumor. Field of view is 2×2 mm.

Four different time points in the evolution of the malignant mass are considered in detail, namely 6, 12, 18 and 24 days post inception. Representative panels (2×2 mm) depicting the tumor evolution over time are presented in **Figure 2**. The pre-existing vessels are laid out in a regular grid with vessels located every 250 µm along each dimension (brown lines in **Figure 2**) establishing normoxic conditions within the surrounding tissue cross-section, as demonstrated previously [17], [19]. At time zero, an avascular tumor nodule of radius ∼50 µm is placed in the center of the regular grid. With time, the nodule grows and develops three identifiable regions: the viable tissue developing at the front of the expanding mass (red); the necrotic tissue located deeper inside the tumor (brown); and the hypoxic tissue intermediately located between the viable and necrotic areas (blue). Also, irregular vessels are seen to sprout from the normal vessels in response to a net balance of pro-angiogenic factors produced by the hypoxic tissue within the tumor. **Figure 2** shows the progressive enlargement of the malignant mass with a continuous growth of the volume ratio associated with the necrotic and hypoxic tissues, as well as of the tumor vascular density.

### Analysis of Vascular Characteristics

**Figure 3:**
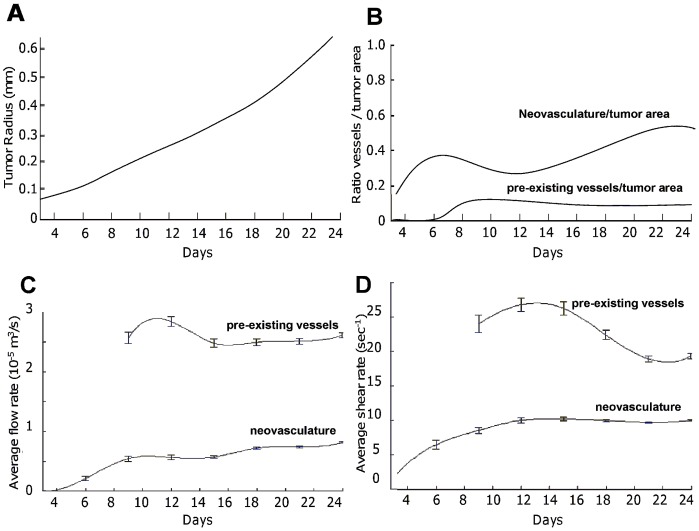
Simulated tumor and vasculature growth presented as a function of the number of days post tumor inception. (A) Tumor radius; (B) blood area fraction, defined as ratio of the vasculature to the tumor area, (C) average vascular flow rate; and (D) average wall shear rate. In (C) and (D), bars denote SEM (standard error of the mean) values estimated over the number of vessel segments in the tumor area (e.g., on day 24, the numbers were 6677 new vessel and 917 pre-existing vessel segments).

The variation of the tumor average radius with time is shown in **Figure 3a** up to 24 days post inception. The tumor shows overall a ∼30 fold increase in radius from ∼0.08 to 0.62 mm. The blood area fraction is introduced as the ratio between the total area covered by the vessel network and the tumor area in a cross section. The variation of such a fraction over time can be readily estimated processing the data of **Figure 2**. This is shown in **Figure 3** for both the pre-existing and neovasculature. Interestingly, for the new vascular network originating with the tumor, the blood area fraction grows with time reaching a value close to 0.6 at 24 days, implying that more than 50% of the tumor cross section is covered by blood vessels. On the other hand, the blood area fraction for the pre-existing vasculature steadily decreases over time being always smaller than 10%. This curve starting at day 6 and rising through day 9 also shows that it takes almost 10 days for the nascent tumor to co-opt the existing vessels that surround it. The average flow rate and wall shear rate within the pre-existing vessels and the neovasculature are shown in **Figure 3c and 3d**, respectively. For the pre-existing vessels, a minor variation is observed over the 24 days which is contained within 10–20% of the corresponding mean values, 2.5×10^−5^ m^3^/s and 23 s^−1^. Differently, these two hydrodynamic parameters change dramatically for the neovasculature starting from zero during the avascular phase of the tumor, growing rapidly over the first 10 days, and reaching 0.8×10^−5^ m^3^/s and 10 s^−1^, respectively, at 24 days. Note that these values are comparable with those observed for the pre-existing vasculature, implying that after 10 days the tumor neovasculature is fully functional.

### Flow Rate and Fraction of Adhering Particles

**Figure 4:**
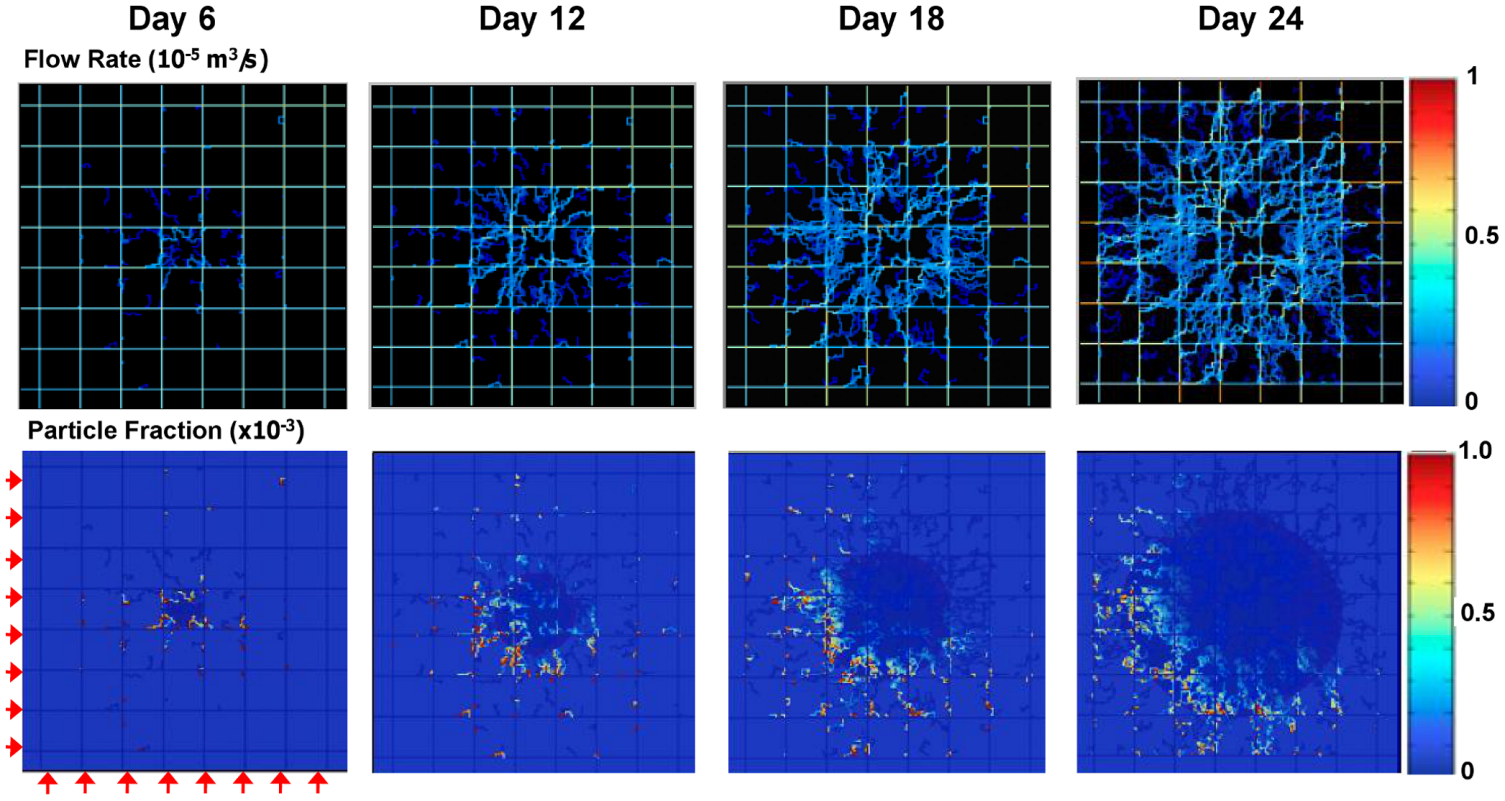
Blood flow rate and adhering particle fraction. The simulated blood flow rate mapped directly over the tumor and pre-existing vascular network (top). The color map is scaled by the maximum flow rate reached in the pre-existing vessels inside the tumor (10^−5^ m^3^/s). The fraction of injected 1,000 nm NPs (×10^−3^) adhering firmly at the blood vessel walls is also shown at ∼100 min after systemic injection (**bottom**). The images correspond to the tumor stages depicted in **Figure 2**. Red arrows indicate the points of injection for the NPs, located upstream with respect to the tumor mass. The parameter *α* is 10^12^ m^−2^ in the tumor neovasculature and 10^10^ m^−2^ in the pre-existing vessels. The parameter *β* is fixed and equals 10^−3^ m^−2^ s. Note that under these conditions, the NPs accumulate mostly at the periphery of the tumor immediately downstream of the injection sites.

Based on these model outputs, it is reasonable to argue that the accumulation of systemically injected NPs within the tumor vasculature would vary with the development stage of the tumor. Referring to the four timepoints considered so far, the distribution of the flow rate and the accumulation of 1,000 nm particles within the tumor vasculature are shown in **Figure 4**. The timescale for nanoparticle binding (assuming instantaneous attachment) is the flow time scale (sec^−1^). The flow rate appears to be relatively constant over time, with a slight increase towards the later stages, and mostly uniform within the malignant mass. As the tumor grows larger beyond the timespan simulated here, this uniformity is expected to be less pronounced. The flow rates are scaled by the flow rate in the pre-existing vessels inside the tumor, as shown by the color map. The NPs are injected upstream of the malignant mass (**Figure 4b** - red arrows), transported by the blood flow and adhere firmly to the vessel walls depending on the local hydrodynamic and biophysical conditions. In particular, for the simulations presented in **Figure 4b**, the parameters *α* and *β* are kept constant and equal to *α* = 10^12^ m^−2^ in tumor-induced vessels and *α* = 10^10^ m^−2^ in the pre-existing vessels, while *β* = 10^−3^ m^−2^ s. The difference in the value of *α* between the pre-existing vessels and the neovasculature reflects the over-expression of specific receptor molecules on the tumor endothelium. Under these hydrodynamic and biophysical conditions, the NPs preferentially deposit at the periphery of the tumor, closer to the injection sites (*tumor inlet*). Indeed, the NP distribution appears to be less uniform as the size of the tumor, and corresponding vasculature, increases. Also moving from the sites of injection towards the center of the malignant mass, the fraction of accumulating particles decreases progressively (from red to blue as indicated by the color map). Although the particles that adhere tend to preferentially bind closer to the injection sites, many particles still pass through the tumor without adhering as can be measured from the fraction of injected particles (**Figure 4**, bottom row). This implies that under these conditions, the majority of the injected NPs that adhere avidly bind to the neovasculature at the tumor inlet and only very few NPs actually adhere deeper into the malignant mass. This computational test demonstrates that high vascular affinity would impair the uniform accumulation of particles within the tumor vasculature. In this initial implementation, we make the simplifying assumption to neglect the effects of recirculation, which may not be negligible.

### Analysis of Particle Vascular Affinity

**Figure 5:**
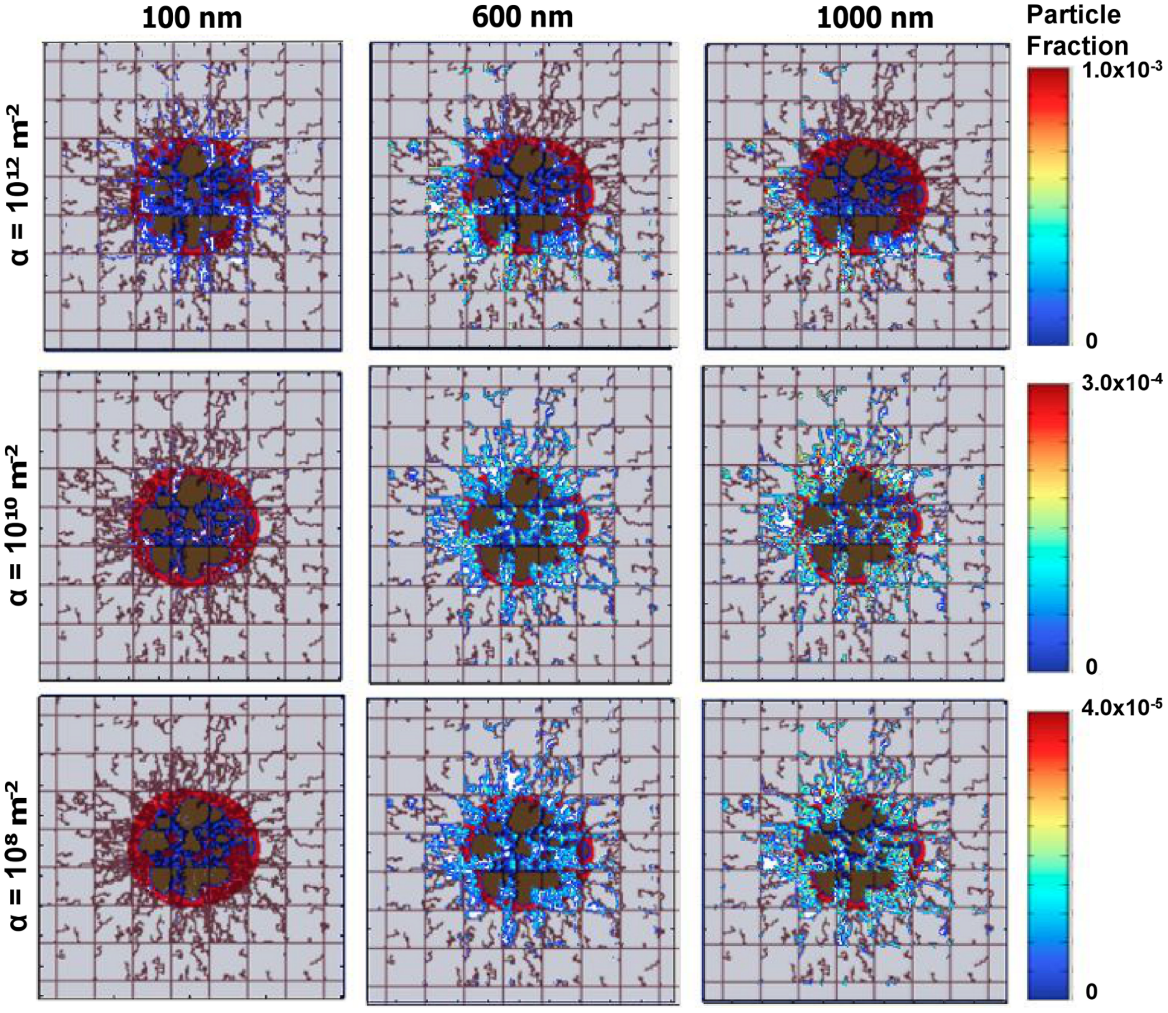
**Fraction of the injected NPs adhering firmly at the blood vessel walls at about 100 min after injection on day 18, upstream of the tumor mass as indicated by the red arrows in** **Figure 4**. The colors on the right provide a measure of the particle fraction adhering mainly to the neovasculature (irregular lines). The columns are related to three different NP sizes, namely 100, 600 and 1,000 nm; whereas the three rows are related to three different values for the parameter *α* (*α* = 10^12^ m^−2^ top row; *α* = 10^10^ m^−2^ middle row and *α* = 10^8^ m^−2^ bottom row). For all cases, *β* = 10^−4^ m^−2^ s while *α* for the pre-existing vessels is 100 times smaller than for the corresponding tumor-induced neovessels. Tumor colors are as in **Figure 2**.

The importance of the NP vascular affinity is more clearly shown in **Figure 5**. Here, NPs with three different sizes, namely 100 nm (left column), 600 nm (middle column) and 1,000 nm (right column), are injected at day 18 in tumors exhibiting different levels of vascular receptor expressions in the neovasculature, namely *α* = 10^12^, 10^10^ and 10^8^ m^−2^. In the pre-existing vessels, *α* is 100 times smaller than in the corresponding neovasculature. For the 100 nm particles, vascular accumulation occurs quite uniformly over the whole neovasculature but it lowers significantly as *α* decreases. For the larger 600 and 1,000 nm particles, the vascular accumulation is quite uniform only for the lower values of the parameter *α*, namely 10^10^ and 10^8^ m^−2^. Differently, at *α* = 10^12^ m^−2^, the larger particles distribute not uniformly with a higher accumulation at the periphery of the malignant mass near the injection site, as seen in **Figure 4**. This computational analysis demonstrates that NP accumulation occurs uniformly throughout the tumor only for moderate vascular affinities.

### Particle Fraction Adhering per Tumor Area

**Figure 6:**
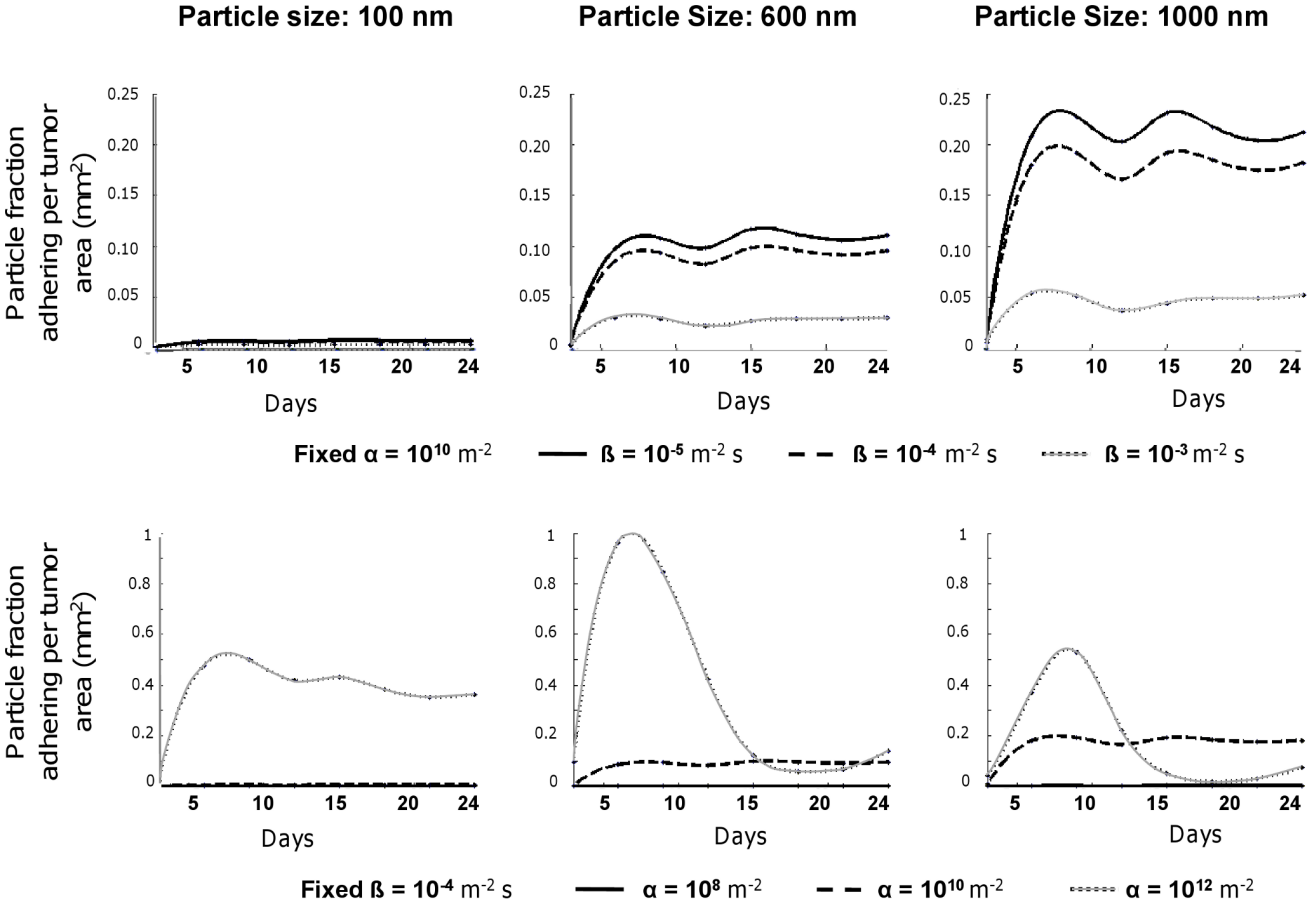
Fraction of the injected NPs adhering firmly at the blood vessel walls per tumor area (mm^−2^) as a function of the tumor development stage (days post tumor inception) for a range of magnitudes of the parameters α and β. **Top:** Fixed *α* = 10^10^ m^−2^ with *β* varying with values of 10^−5^ m^−2^s (solid black line), 10^−4^ m^−2^s (dashed black line), and 10^−3^ m^−2^s (solid/dotted gray line). **Bottom:** Fixed *β* = 10^−4^ m^−2^s with *α* varying with values of 10^8^ m^−2^ (solid black line), 10^10^ m^−2^ (dashed black line), and 10^12^ m^−2^ (solid/dotted gray line).

**Figure 6** provides the fraction of NPs compared to the injected dose adhering per unit surface at the tumor vasculature as a function of the particle diameter *d*, parameters *α* and *β*, and stage of tumor development. For a fixed *α* = 10^10^ m^−2^ (**Figure 6**– top row), the number of NPs accumulating in the tumor vasculature grows with time rapidly over the first 10 days and then levels out for longer time points. This is consistent with the behavior of the average flow rate and shear rate in the neovasculature (**Figure 3**) showing a rapid increase within the first 10 days post tumor inception. The same plot shows that an increase in *β* leads to a reduction of the number of particles accumulating within the tumor vasculature, and the opposite behavior is depicted for the NP size *d*. This can be interpreted by considering that as *β* increases the contribution of the dislodging hydrodynamic forces increases, whereas, for these specific conditions, an increase in *d* is accompanied by an overall increase in adhesive strength. Note that non-specific accumulation in the pre-existing vasculature is negligibly small due to the lower affinity (100 times) and higher average shear rate (2–4 times).

The variation with *α* of the fraction of adhering NPs presents a more complex and interesting behavior (**Figure 6**– bottom row). For *α* = 10^12^ m^−2^, the accumulation of the 100 nm particles grows rapidly over the first 10 days and then reduces slowly up to 24 days. Differently, for the 600 and 1,000 nm particles, the fraction of adhering particles reaches a maximum at about 10 days and then decreases rapidly to almost zero up to 24 days. For lower values of *α* (10^8^ and 10^10^ m^−2^), the behavior is similar to that observed in **Figure 6** (top row). These results emphasize the importance of properly modulating the size of the NPs, *d,* with respect to the adhesion parameter *α* to foster tumoritropic accumulation and a uniform distribution of the NPs.

For the conditions considered here (**Figure 6**), after the first 10 days, the fraction of NPs accumulating within the tumor vasculature is quite constant over time and uniform in space only for *d* = 100 nm and *α* = 10^12^ m^−2^, and for *d* = 600 and 1,000 nm and *α* = 10^10^ m^−2^. At 24h, the fraction of accumulating particles is about 0.4 mm^−2^ for *d* = 100 nm and *α* = 10^12^ m^−2^; about 0.2 mm^−2^ for = 1,000 nm and *α* = 10^10^ m^−2^; and 0.1 mm^−2^ for *d* = 600 nm and *α* = 10^10^ m^−2^. Note that smaller NPs (100 nm) require a much larger *α* as compared with the larger NPs to provide similar levels of uniform distributions and particle accumulation doses. Indeed, the larger 600 and 1,000 nm NPs exhibit a payload total volume about 100 to 1,000 times higher than the smaller 100 nm NPs.

## Discussion

A two-dimensional model for the growth of a vascularized tumor is integrated with a mesoscale formulation for the vascular adhesion of systemically injected NPs. The former allows for estimating the evolution with time of the tumor microvasculature, while the latter quantifies the number of NPs adhering to the vessel walls. The NP accumulation is estimated as a function of the surface density of vascular receptors and NP ligands; ligand-receptor molecular affinity, and diameter of the spherical NPs.

### The Effect of Vascular Characteristics on Particle Accumulation

As the tumor grows over time, new vessels are formed within the malignant mass. The average flow rate and shear rate in the tumor neovasculature grows rapidly within the first 10 days, reaching almost the same values as in the pre-existing vessel network. The ratio between the area occupied by the new vessels and the tumor mass increases over time reaching a value of ∼40% at day 10 and ∼60% at day 24 (**Figure 3**). The accumulation of systemically injected NPs is consistent with the development of the neovasculature within the tumor mass. Regardless of the particle properties (size, type and surface density of ligands) and vascular features (surface density of receptors), the particle accumulation grows rapidly reaching a maximum value at ∼10 days post tumor inception. At earlier stages, the average flow rate in the tumor is insufficient to support the accumulation of any circulating agents, either NPs or small molecules, and most them are inevitably transported away through the regular, pre-existing vasculature characterized by a higher flow rate [24].

### The Role of Vascular Affinity

Vascular affinity affects both the distribution and the total accumulation within the tumor of the NPs, as shown in **Figures 5** **and** **6**, respectively, especially through the variation of the parameter α, which is mainly related to the affinity of particles for vessels. The NP affinity for the tumor endothelial cells is regulated by the particle size (diameter *d*), and the surface density of ligands and endothelial receptors and their molecular affinity to each other. High vascular affinity leads to particles depositing mostly at the tumor inlet vessels leaving the core and outlet vessels depleted of NPs. On the other hand, low *d* affinities facilitate a more uniform distribution of the injected NPs throughout the tumor. By using ανβ3-targeted nanoparticles, non-uniform distributions within solid tumors have been observed in agreement with our theoretical results, with the nanoparticles accumulating primarily at the tumor periphery and in vessels with more active angiogenic progression [25], [26]. A reduction in vascular affinity is also accompanied by a decrease in the overall NP tumor accumulation. The computational model enables identifying the proper balance between NP tumoritropic accumulation and uniform distribution.

### Model Robustness

In the simulations, we run the tumor mass up to the time of treatment and stop its evolution to inject the particles. For the same set of parameters, the model outputs the same evolution of the tumor mass. Over multiple simulation runs (as we have done previously, e.g., [19]), we consistently find that the flow rate, vascular density, and shear rates are very similar at the respective time points. Consequently, the particle fraction adhering per tumor area does not really differ from the results shown in **Figure 6**. Even though the simulations are stochastic, tumors grown under these conditions are similar, and so are their flow rate and vascular densities.

### Conclusion

A multidimensional computational model has been developed to predict the vascular accumulation of systemically injected NPs in the tumor neovasculature. It is predicted that the fraction of NP accumulating in the malignant tissue depends on the stage of the disease (vascularity and expression of endothelial receptors), and that a moderate NP vascular affinity provides the proper balance between optimal spatial distribution and absolute tumoritropic accumulation.

## Methods

### 1.0. Modeling the Tumor

**Figure 7:**
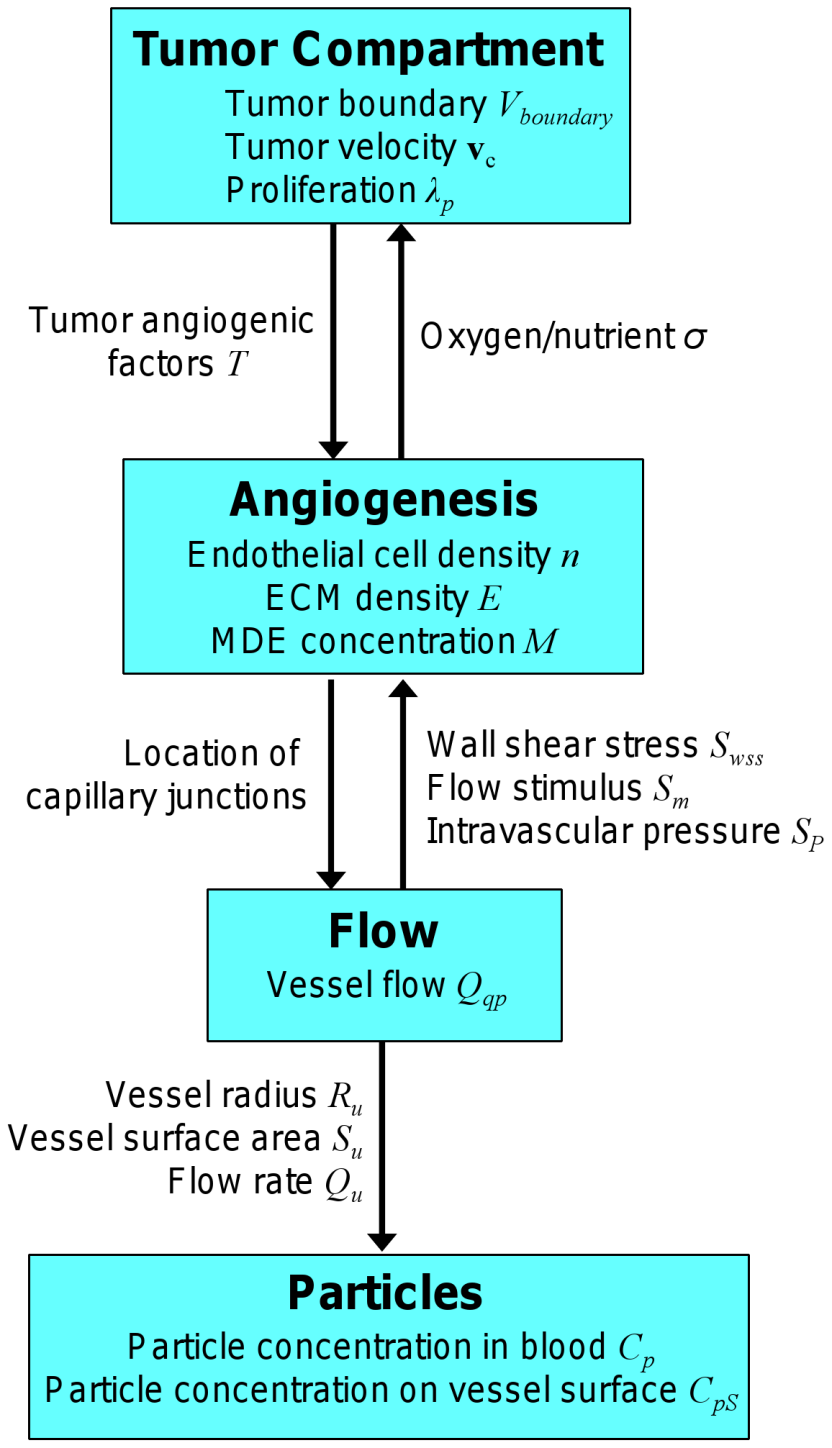
Model overview showing the main components, variables, and key system interactions.

**Figure 8:**
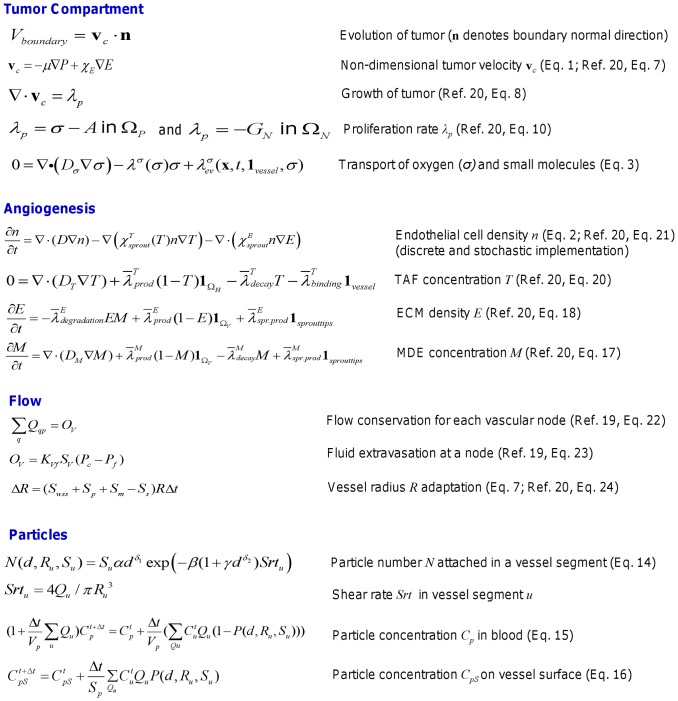
Main equations and variables related to the model system components.

**Figure 7** provides an overview of the main model components and **Figure 8** summarizes the main equations. Since the spatiotemporal dynamics of solid tumor growth depend upon the balance of proliferation, apoptosis, necrosis, migration, and cell-cell and cell-ECM adhesion [27], [28], we express these dynamics in a mathematical model by physical conservation laws acting on the cells and tissue [19]–[20]. These laws represent conservation of mass (due to cell creation and destruction), conservation of momentum (due to tissue velocity as the tumor grows or shrinks), and physical transport (diffusion, advection, and convection of substances). Angiogenesis is incorporated by coupling models of vessel growth, branching, and anastomosis [29], together with blood flow [30]. The tumor vasculature acts as a source of oxygen and cell nutrients, as well as nanoparticles. This enables evaluation of the local effects of vascularization and blood flow on tumor cells and nanoparticle transport, and provides a better understanding of the micro-environment conditions such as hypoxia that lead to the development of intra-tumor heterogeneity.

The computational model describes in a 2-D Cartesian coordinate system the viable and necrotic tumor tissue, diffusion of small molecules (cell substrates and particles), and conservation of mass and momentum (as detailed in [19]). The initial condition is a small tumor (<50 µm diameter) in the middle of the pre-existing vasculature grid, as shown in **Figure 2**. Mass conservation equations describe growth (proliferation as a function of total cycling cells) and death from hypoxia (necrosis as a function of oxygen). These are combined with diffusion of small molecules to a reaction-diffusion equation. Rate constants for proliferation and apoptosis depend on the availability of cell nutrients and oxygen and are thus spatiotemporally heterogeneous. Model parameters values are calibrated to published experimental data as in [20], [28], [31]–[33]. The main tumor model parameters are summarized in Wu et al. (2013) [19].

**1.1. Tumor growth.** Following [20], we denote the tumor mass as 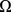 and denote its boundary as 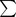. The tumor is divided into three regions: a proliferating region 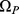 where the tumor cells have sufficient levels of oxygen and cell nutrients for proliferation; a hypoxic region 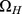 where the oxygen and cell nutrient levels are sufficient for the cells to survive but not enough to sustain proliferation; a necrotic region 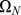 where the nutrient level is insufficient to maintain the cells alive. The non-dimensional tumor velocity is given by a generalized Darcy’s law [20]:
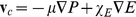
(1)where μ is the cell-mobility modeling the net effects of cell-cell and cell-matrix adhesion, *P* is the oncotic pressure, χ*_E_* is the haptotaxis coefficient, and *E* is the ECM density (composed of a non-diffusible matrix macromolecules such as fibronectin or collagen). Details for χ*_E_* and *E* are in [19]–[20]. We associate the growth of the tumor with the rate of volume change by assuming that the density of cells is constant in the proliferating region, 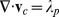, where 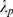 is the non-dimensional net proliferation rate 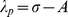 in 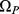 and 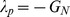 in 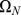 (*A* is the natural apoptosis rate and *G_N_* is the non-dimensional rate of volume loss in the necrotic regions, assuming that fluid is removed and cellular debris is constantly degraded; σ is the concentration of oxygen and cell nutrients). Note that a Gompertzian growth curve [34]–[36] of the form 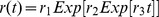 can be fitted to the numerical data shown in **Figure 3a** by using *r_1_* = 0.85, *r_2_* = −4. 0, and *r_3_* = −0.1.

#### 1.2. Angiogenesis

To account for tumor-induced angiogenesis, we couple the tumor growth model with an angiogenesis model inspired by McDougall et al.(2006) [30] that accounts for blood flow through the vascular network, non-Newtonian effects, vascular leakage and vascular network remodeling due to wall shear stress and mechanical stresses generated by the growing tumor. The angiogenesis model is described in detail in [19]–[20]. Briefly, the model assumes that endothelial cells are stimulated to migrate based on chemotaxis due to tumor angiogenic factors (TAF) released by tumor hypoxic tissue and haptotaxis due to gradients of extra-cellular matrix (ECM), as well as random motility. The non-dimensional equation describing the conservation of endothelial cells is [20]:
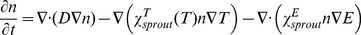
(2)where *n* is the non-dimensional endothelial cell density per unit area, and *T* and *E* are the TAF and ECM concentrations, respectively. The diffusion (random migration) coefficient *D* is assumed constant, while the chemotactic and haptotactic migration are described by 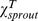 and 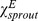, respectively [20]. The displacement of individual endothelial cells, occurring at the tips of growing sprouts, is given by the discretized (and stochastic) form of **Eq. (2)** [20].

For the blood flow we specify an inflow and an outflow pressure (as in [19]). As the tumor grows due to cell proliferation, it remodels the surrounding vessels and leads to the creation of new vessels due to the secretion of a net balance of angiogenic factors from hypoxic tumor cells.

#### 1.3. Transport of oxygen and small molecules

We use a model [19] following previous work [20] that describes the transport of small molecules *s* such as oxygen (*s* = *σ*) at the point of release from the vasculature by quasi-steady reaction-diffusion equations (we assume that the timescale for cell proliferation is much larger (∼1 day) than the timescale for the diffusion of small molecules (∼1 min or less)). We assume that the small molecules are supplied by the pre-existing vasculature as well as the neo-vasculature at rates 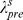 and 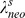, respectively, diffuse into the normal and cancerous tissue with a constant diffusion coefficient *D_s_*, are uptaken both by the normal cells (with rate 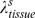) and tumor cells (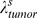 in the proliferating region and *q_s_* in the hypoxic region), and decay (with rate 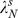) in the necrotic regions. The equations are:
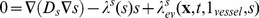
(3)

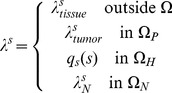
(4)where in general *q_s_* is a smooth interpolating function that matches 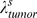 between the proliferating and hypoxic tumor regions, and also matches 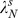 between the hypoxic and necrotic tumor regions; **x** is position in space; *t* is time and **1** is the characteristic function of the vessels (i.e., **1**_vessel_ equals 1 at the locations of the vessels and 0 otherwise). In the case of oxygen (*s* = *σ*) the source term is:
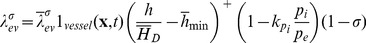
(5)where 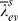 is the constant transfer rate from the vasculature (for simplification, assumed to be the same for both pre-existing and tumor-induced vessels),and h is the hematocrit in the neo-vascular network related to oxygen extravasation (following [20]). This extravasation is affected by the interstitial pressure pi outside of the vessels scaled by the effective pressure p_e_ (the blood pressure minus the transcapillary osmotic pressure difference), with 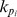 representing the weight of the convective transport component of the small molecules across the vessel wall. The constants 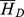 and h_min_ represent the normal blood hematocrit and the minimum hematocrit required for oxygen extravasation, respectively. The conditions at all boundaries for the diffusion equations (as well as the pressure and angiogenic factors) are taken to be zero Neumann condition,
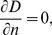
(6)where 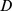 is the element at the boundary (either oxygen, pressure, or angiogenic factors).

#### 1.4. Vessel radius adaptation

The computation of vessel radii is based on [19]–[20], [30], [37]–[38]. We set the initial value of all radii to be 6 µm (as in [20]). The variation of the radius Δ*R* depends on the wall shear stress, the intravascular pressure, and the blood flow carrying the hematocrit, characterized as follows:
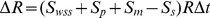
(7)

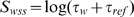
(8)

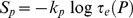
(9)

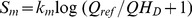
(10)


*Swss* is the stimulus by the wall shear stress *τ_ω_* while *τ_ref_* is a constant included to avoid singular behavior at low shear rates; *τ_ω_* is calculated from [37], [39]:
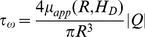
(11)where 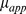 is the apparent viscosity. |*Q*| is the absolute value (mode) of the flow rate. *S_p_* is the stimulus by the intravascular pressure *P* in the form:
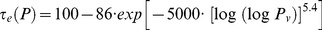
(12)where the values are obtained from [20], [30], [37]–[38]. Sm is the stimulus by the flow Q carrying hematocrit HD, where Q_ref_ is a reference flow assumed to be larger than most other flows in the network. The parameters kp and km are the intensity coefficients, while S_s_ is related to the tendency of the vessel to shrink under tissue pressure [19].

### 2.0. Modeling the Nanoparticle Accumulation

The vascular accumulation of blood-borne NPs is mediated by the fine regulation between dislodging hydrodynamic forces and adhesive interactions arising at the particle-cell interphase. The latter can be specific interactions associated with the formation of stable ligand-receptor molecular bonds or non-specific interaction such as those related to colloidal forces (van der Waals, electrostatic and steric). The formation of molecular bonds at the particle-cell interphase can be described as a chemical reaction with forward and reversing rates (binding and unbinding rates for the ligand-receptor couple), where the reaction rates are influenced by the external forces exerted over the particle. A probability of adhesion *P_a_* can be introduced to quantify the strength and likelihood of firm adhesive interactions between a NP decorated with ligand molecules and a cell membrane expressing specific counter molecules (receptor molecules) [15]. *P_a_* depends on the NP properties – size, shape and surface density of ligands – and local vascular biophysical conditions – wall shear rate, and surface density of receptors. For spherical particles, the number *n* of particles with diameter *d* adhering within a blood vessel with shear rate *S* can be written as [15]:
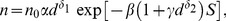
(13)where *n_o_* is the number of particles exposed to the vessel walls and the parameters *α*, *β* and *γ* are, respectively, proportional to *i*) the surface density of receptors on the endothelial cells (*m_r_*) and ligands on the particle (*m_l_*), and the ligand-receptor affinity under zero external force (*K_A_^0^*) (*α ∝ m_r_ m_l_ K_A_^0^*); *ii*) the characteristic length scale of the ligand-receptor bond (*χ*) and the viscosity of water (*μ*) (*β ∝ χ μ*/(*k_B_T m_r_*), where *k_B_T* is the Boltzmann thermal energy); and *iii*) the inverse of the surface density of receptors. The coefficients *δ*_1_ (∼0.45) and *δ*_2_ (∼1.57) are derived from the best fit of **Eq. (13)** with the experimental data shown in Boso and colleagues [24]. For typical values of *m_r_* = 10^12^ #/m^−2^; *m_l_* = 10^14^ #/m^−2^ and *K_A_^0^* = 10^−14^ m^2^, the parameter *α* = *O*(10^12^) m^−2^ [15], [40]. For lower ligand-receptor affinities, *α* is correspondingly lower. For typical values of *m_r_* = 10^12^ #/m^−2^, *χ* = 10^−10^ m^−1^ and *μ = *10^−3^ Pa s^−1^, the parameter *β* = *O*(10^−4^) m^−2^ s. The parameter *γ* = *O*(10^4^) m^−*δ*2^. A uniform concentration of NPs in the blood is assumed, with the maximum normalized to 1. Due to heterogeneities in the vascular flow, a heterogeneous spatio-temporal distribution of the particles within the tumor vasculature is also expected.

#### 2.1. Particle delivery

We multiply both sides of the simplified relationship in **Eq. (13)** by *S_u_* = 2π*R_u_L_u_*, which is the surface area of each vessel segment, where *R_u_* and *L_u_* are the radius and length of the vessel, respectively. The particle number *N* attached in each vessel segment is obtained by:
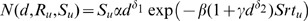
(14)where the shear rate is 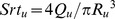 and *Q_u_* is the flow rate (see below). Here we can vary the values of *α, β* and *γ*. We can vary the vascular radius shrinking tendency parameter *ks* by itself to evaluate the change in *S_u_* and *Srt_u_*. We can also vary the pressure drop along the vessel segment to change the value of *Q_u_*.

#### 2.2. Particle concentration

For the particle concentration *Cp* (fraction per m^3^) in the blood and on the vessel surface *CpS*, we specify the mass conservation equations in the vessels and on the vessel surface:
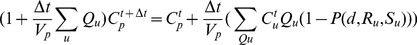
(15)

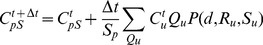
(16)where the *u*’s represent the upstream neighbor nodes and *Qu*’s are the flow rates from nodes *u*’s to node *p*. In **Eq. (15)**, the change in particle concentration in the blood is due to what flows in (left-hand side) and what flows out (first term, right-hand side) and what adheres (second term, right-hand side). The change in particle concentration on the surface (**Eq. (16)**, left hand side) depends on the amount that flows in (first term, right hand side), plus the amount that adheres (second term, right hand side). The parameters 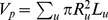 and 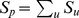 represent the overall volume and surface area, respectively, for the vessel segment from all the upstream *u*’s to *p*. The concentration *CpS* accumulates from *Cp* over the treatment time (e.g., from the injection time to the time when the particles in the blood are convected out of the system). The fraction of particles attached to the surface of each vessel segment is calculated as *MpS = SpCpS*.
